# 588. The Association Between Contact Precautions for Methicillin-resistant *Staphylococcus aureus* (MRSA) Colonization and MRSA Bloodstream Infection (BSI) Rates in Los Angeles County Hospitals: Results of a Countywide Survey

**DOI:** 10.1093/ofid/ofae631.183

**Published:** 2025-01-29

**Authors:** Pam Lee, Kelsey OYong, Zachary A Rubin, Loren G Miller, Deborah Kupferwasser

**Affiliations:** Harbor UCLA, Long Beach, California; Los Angeles County Department of Public Health, Los Angeles, California; Los Angeles County Department of Public Health, Los Angeles, California; Lundquist Institute at Harbor-UCLA Medical Center, Los Angeles, CA; Division of Infectious Diseases, the Lundquist Institute at Harbor-UCLA Medical Center, Torrance, CA, Torrance, California

## Abstract

**Background:**

Contact precautions are frequently used to prevent in-facility transmission of MRSA, but their efficacy has been questioned. We hypothesized that contact precautions use would not correlate with MRSA bloodstream infection (BSI) rates in LA County hospitals.

**Methods:**

The LA County Department of Public Health conducted a survey of LA County acute care hospitals’ use of contact precautions for MRSA colonization, history of MRSA infection/colonization, and active MRSA infection with or without uncontained purulence. We modeled National Healthcare Safety Network (NHSN) reported healthcare-associated MRSA BSI rates from 2022-23 via a negative binomial multivariable regression model. We controlled for hospital type (general acute care hospitals vs. long-term acute care hospitals (LTACHs)), number of intensive care unit (ICU) beds, and MRSA BSI admission prevalence rates.

**Results:**

Of the 82 acute care hospitals in LA County, 69 completed the survey (84% response rate). MRSA contact precaution use was stratified into three groups: Least strict (no contact precautions or contact precautions only for active infection with uncontained purulence, 24% of hospitals), intermediate (contact precautions for any active infection, 32% of hospitals), and most strict (contact precautions for >2 reasons, 44% of hospitals).

We found higher MRSA BSI in LTACHs vs. hospitals (Incidence rate ratio [IRR] 3.50, 95% CL 1.80-6.80, p = 0.0002), but no relationship with ICU beds (IRR 1.00, 95% CL 0.99-1.01, p = 0.15), MRSA BSI admission prevalence rates (IRR = 4.40, 95% CL 0.30-63.10, p = 0.27), or contact precaution use (IRR 0.80, 95% CL 0.50-1.30, p = 0.29 for the least strict group; IRR 1.40, 95% CL 0.89-2.20, p = 0.14 for the intermediate group, both in comparison to the most strict group).

**Conclusion:**

Our survey of hospitals across LA County, which has a population of ∼10 million, found no significant association between stringency of MRSA contact precaution use and healthcare-associated MRSA BSI rate. Our findings support other data that fail to find clinical benefit to contact precautions for MRSA colonization/infection. Considering potential downsides of contact precautions for MRSA, the assumption that MRSA requires contact precautions may need to be reconsidered.

**Disclosures:**

**Loren G. Miller, MD MPH**, Armata: Grant/Research Support|Contrafect: Grant/Research Support|GSK: Grant/Research Support|Merck: Grant/Research Support|Paratek: Grant/Research Support

